# Heat-Treated Ni-Coated Fibers for EMI Shielding: Balancing Electrical Performance and Interfacial Integrity

**DOI:** 10.3390/polym17121610

**Published:** 2025-06-10

**Authors:** Haksung Lee, Man Kwon Choi, Seong-Hyun Kang, Woong Han, Byung-Joo Kim, Kwan-Woo Kim

**Affiliations:** 1 Reclaimed Land Agricultural Research Center, National Institute of Crop and Food Science, RDA, Wanju 55365, Republic of Korea; lhs0221@korea.kr; 2 National Institute of Horticulture and Herbal Science, RDA, Haman 52054, Republic of Korea; choimk82@korea.kr; 3 Industrialization Division, Korea Carbon Industry Promotion Agency, Jeonju 54852, Republic of Korea; rkdtjdgus95@gmail.com (S.-H.K.); shareyi@kcarbon.or.kr (W.H.); 4 Department of Carbon Materials and Fiber Engineering, Jeonbuk National University, Jeonju 54896, Republic of Korea; 5 Department of Materials Science and Chemical Engineering, Jeonju University, Jeonju 55069, Republic of Korea

**Keywords:** electroless nickel plating, EMI shielding, post-heat treatment, interfacial shear strength, conductive fiber-reinforced composites

## Abstract

With the growing integration of electronic systems into modern infrastructure, the need for effective electromagnetic interference (EMI) shielding materials has intensified. This study explores the development of electroless Ni-plated fiber composites and systematically investigates the effects of post-heat treatment on their electrical, structural, and interfacial performance. Both carbon fibers (CFs) and glass fibers (GFs) were employed as reinforcing substrates, chosen for their distinct mechanical and thermal characteristics. Ni plating enhanced the electrical conductivity of both fibers, and heat treatment facilitated phase transformations from amorphous to crystalline Ni_3_P and Ni_2_P, leading to improved EMI shielding effectiveness (EMI-SE). NGF-based composites achieved up to a 169% increase in conductivity and a 116% enhancement in EMI-SE after treatment at 400 °C, while NCF-based composites treated at 800 °C attained superior conductivity and shielding performance. However, thermal degradation and reduced interfacial shear strength (IFSS) were observed, particularly in GF-based systems. The findings highlight the importance of material-specific thermal processing to balance functional performance and structural reliability. This study provides critical insights for designing fiber-reinforced composites with optimized EMI shielding properties for application-driven use in next-generation construction materials and intelligent infrastructure.

## 1. Introduction

With the rapid advancement of electronic systems, wireless communication networks, and embedded sensor technologies, modern civil infrastructure is increasingly integrating intelligent functionalities to enhance structural monitoring, energy efficiency, and real-time health assessment. However, the growing density of electronic components embedded within structural elements introduces critical challenges related to electromagnetic interference (EMI). EMI can cause signal degradation, sensor malfunction, and disruption of communication networks. Consequently, there is a growing demand for the integration of electromagnetic shielding materials into construction systems to ensure electromagnetic compatibility (EMC) in civil structures [1,2,3,4,5,6,7,8,9,10,11,12]. EMI shielding refers to the attenuation of electromagnetic waves through reflection and/or absorption by shielding effectiveness (SE) [13,14,15,16,17,18,19].

Traditionally, metals such as copper (Cu), nickel (Ni), and aluminum (Al) have been widely used as EMI shielding materials due to their excellent electrical conductivity. However, these materials pose significant limitations in large-scale construction, including high weight, susceptibility to corrosion, and elevated cost [20,21]. As an alternative, polymer-based shielding materials have gained attention due to their lightweight and ease of processing. Nevertheless, the inherently low conductivity of most polymers restricts their EMI shielding performance. To overcome this limitation, recent research has focused on the incorporation of conductive fillers into polymers. Concurrently, interest has also grown in developing electrically conductive cementitious composites, which combine EMI shielding functionality with the structural integrity of concrete.

Among various conductive reinforcements, carbon-based materials, such as carbon nanotubes (CNTs), graphene, and carbon fibers (CFs), have shown promising EMI shielding properties due to their exceptional conductivity and low density [22,23]. Despite these advantages, their widespread application in construction is hindered by high costs, dispersion difficulties in cement matrices, and scalability issues [14,21,24,25,26,27,28,29]. As an alternative, glass fibers (GFs) offer a high aspect ratio, mechanical durability, and cost-effectiveness, making them a practical reinforcement option. However, due to their insulating nature, GFs require surface modification to be viable for EMI shielding. One of the most effective techniques is electroless metal plating, which allows uniform coating deposition, scalability, and cost-effectiveness. Among various metallic coatings, nickel (Ni) is widely studied for its high conductivity, corrosion resistance, and structural integrity in composite systems.

Recent research has focused on enhancing EMI shielding effectiveness in cementitious matrices by incorporating conductive fillers and metal-coated reinforcements [30]. For example, Park et al. demonstrated that CNT-reinforced cement composites showed improved EMI-SE due to the formation of interconnected conductive networks [31]. Similarly, Tan et al. reported that nickel-coated fibers embedded in composites effectively attenuate electromagnetic waves while preserving mechanical properties, making them ideal for smart infrastructure and shielding enclosures [32].

These studies highlight the potential of metal-coated fibers and conductive additives to improve the functional properties of cementitious materials, paving the way for the development of next-generation EMI shielding concrete composites. Surface modifications such as electroless Ni plating have been particularly emphasized for developing conductive fiber coatings and multilayered shielding structures. These approaches often rely on the dispersion of conductive fillers in the matrix or the application of thin conductive layers to improve surface conductivity and wave attenuation. Lee et al. [33] reported that FeCoNi-plated fiberglass composite achieved EMI-SE values of approximately 37 dB, highlighting the potential of metal-coated GFs. Similarly, Bozzini et al. [34] showed that post-heat treatment of electroless Ni-P coating enhanced wear resistance and long-term durability.

While several studies have explored the effects of heat treatment on metallic coatings, limited attention has been paid to its influence on the microstructure and EMI shielding properties of Ni-coated fibers, particularly NGFs. Kim et al. [35] examined the heat-induced structural transformation in Ni-plated CFs, reporting changes that enhanced conductivity and shielding effectiveness. These studies have provided valuable insights into the behavior of carbon-based reinforcement, particularly in terms of microstructural optimization and performance enhancement. Glass fiber (GFs), which offer advantages such as cost-effectiveness, high mechanical durability, and widespread availability, have also emerged as promising candidates for EMI shielding applications. However, the effects of post-heat treatment on Ni-coated glass fibers (NGFs) have not been thoroughly explored. Given the inherent differences between carbon fibers and glass fibers, including thermal expansion behavior, interfacial bonding characteristics, and structural stability, it remains unclear how heat-induced modifications in Ni layers impact the electrical conductivity, interfacial shear strength (IFSS), and EMI shielding performance of NGFs.

Therefore, this study aims to systematically investigate the effects of post-heat treatment on Ni-coated glass and carbon fibers. By conducting a comparative analysis, this research seeks to elucidate the distinct responses of GF and CF reinforcements to post-heat treatment, ultimately contributing to the advancement of conductive fiber-reinforced composites for EMI shielding applications. The findings of this study will provide valuable insights into the optimization of fiber-reinforced composites for smart concrete and multifunctional construction materials, with potential implications for intelligent infrastructure and electromagnetic shielding cementitious systems.

## 2. Materials and Methods

### 2.1. Materials

E-glass fiber fabric (HD Fiber, Yangsan, Republic of Korea) and polyacrylonitrile (PAN)-based high-strength carbon fibers (CFs) (T-300, 3K, Toray Co., Ltd., Tokyo, Japan) were selected as reinforcing materials due to their distinct mechanical and electrical properties. The matrix material consisted of an epoxy resin (diglycidyl ether of bisphenol-A, YD-128, Kukdo Chemicals & Metals Co., Ltd., Seoul, Republic of Korea) and a curing agent, 4,4′-diaminodiphenylmethane (Tokyo Chemical Industry Co., Ltd., Tokyo, Japan), mixed in a stoichiometric ratio to ensure optimal crosslinking [35].

### 2.2. Electroless Ni Plating

Electroless Ni plating was carried out following a two-step pretreatment process consisting of sensitization and activation. Tin chloride (SnCl_2_) in hydrochloric acid (HCl) was used as the sensitizing agent, while palladium chloride (PdCl_2_) in HCl served as the activating agent. This pretreatment facilitated the formation of Sn/Pd catalytic nuclei on the GF surface, which subsequently promoted nickel deposition during the electroless plating process.

The composition of the electroless Ni-plating bath is provided in Table 1 [36]. Nickel sulfate (NiSO_4_·6H_2_O) and nickel chloride (NiCl_2_·6H_2_O) served as Ni ion sources, while sodium citrate (Na_3_C_6_H_5_O_7_·1.5H_2_O) functioned as a complexing agent, regulating the availability of free metal ions in solution during the reduction reaction. Sodium hypophosphite hydrate (NaH_2_PO_2_·2H_2_O) functioned as the reducing agent, ammonium chloride (NH_4_Cl) was employed as a buffer, and lead nitrate (Pb(NO_3_)_2_) served as a stabilizer.

Following pretreatment, both GFs and CFs underwent electroless Ni plating for varying durations of 1, 3, 5, and 10 min in a plating bath maintained at a pH of 4 and a temperature of 85 ± 2 °C. The resulting Ni-plated GF and CF samples were designated as NGF-T and NCF-T, respectively, where T represents the plating duration.

### 2.3. Post-Heat Treatment and Fabrication

Post-heat treatment was performed to evaluate the thermal effects on the mechanical and electrical properties of Ni-plated glass fibers (NGFs) and carbon fibers (NCFs). The primary objective was to investigate how heat-induced microstructural transformations affect tensile strength, interfacial shear strength (IFSS), and electrical conductivity.

**Figure 1:**
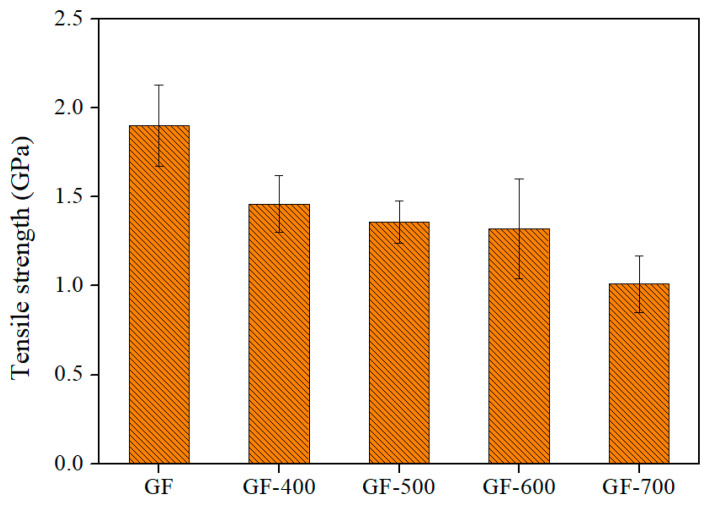
Graph of tensile strength of GF according to temperature change.

The tensile strength of GFs decreases with increases in heat-treatment temperature and time. Their tensile strength was measured as a function of the heat-treatment temperature, as shown in Figure 1. For glass fibers (GFs), a post-heat treatment at 400 °C was conducted, as previous studies have shown that temperatures exceeding this threshold can significantly degrade fiber integrity. The tensile strength of untreated GFs was measured at approximately 1.9 GPa, which decreased to 1.46 GPa after heat treatment, representing a 23% reduction. Despite this decrease, the fiber structure remained intact, indicating that 400 °C is an optimal temperature for enhancing electrical conductivity without compromising structural integrity. The post-heat treatment was performed under N_2_ flow at a rate of 10 °C/min, maintaining the target temperature for 10 min. For carbon fibers (CFs), post-heat treatment was conducted from 500 to 800 °C to assess the thermal stability and microstructural transformation of Ni-coated CF composites. Unlike GFs, CFs can withstand higher temperatures without losing mechanical integrity [35,37].

To fabricate the composite laminates, epoxy resin was mixed with the curing agent DDM (diaminodiphenylmethane) at a stoichiometric ratio of 1:1. The mixture was stirred on a hot plate at 80 °C for 30 min to ensure complete pre-polymerization. The prepared resin was then applied to both NGF and NCF reinforcements using the hand lay-up method. Each specimen was stacked into a three-ply laminate configuration. Following lay-up, the laminates were vacuum-sealed and subsequently hot-pressed at 160 °C under a pressure of 10 MPa for 60 min. The resin-to-fiber weight ratio was maintained at 3:7 throughout the process. The average diameter and density of the glass fibers used were 9 μm and 2.55 g/cm^3^, respectively, while the density of the nickel plating was 8.9 g/cm^3^. The thickness of the nickel coating layer ranged from 0.2 to 0.9 μm, and the weight fraction of the metal-coated reinforcement was fixed at 70 wt%. Based on these parameters, the composite composition by weight was calculated. When the nickel coating thickness was 0.2 μm, the resin/fiber/coating composition was 24.53:57.24:18.23 wt%. At a thickness of 0.9 μm, the composition shifted to 14.43:33.67:51.90 wt%, reflecting the significant influence of plating thickness on the overall composite formulation. The composites were designated as NGF-T/epoxy and NCF-T/epoxy, where T represents the plating duration. Similarly, heat-treated samples were labeled as NGF-T-400/epoxy and NCF-T-800/epoxy to indicate the post-heat treatment conditions.

### 2.4. Surface and Structure Characterization

The surface morphology and structural properties of NGF-T, NGF-T-400, NCF-T, and NCF-T-800 were examined using scanning electron microscopy (SEM, SUPRA40VP, Carl Zeiss, Oberkochen, Germany). To minimize the charging effects, the surfaces were Pt-coated before analysis. The SEM analysis was conducted at a base pressure of approximately 5.0 × 10^−5^ Pa with an acceleration voltage of 15 kV.

SEM images revealed that the surface morphology of Ni-plated GFs became rougher and denser after post-heat treatment, suggesting grain growth and agglomeration of the Ni layer. For Ni-coated CFs, heat treatment at 800 °C induced structural densification and enhanced surface roughness, which contributed to improved conductivity and shielding effectiveness.

To further investigate the crystal structure changes, X-ray diffractometry (XRD, X’pert Powder, Malvern Panalytical, Almelo, The Netherlands) was performed using Cu-Kα radiation at a scan rate of 2°/min over a 2θ range of 10–80°. The XRD results demonstrated that NGF-T samples exhibited a broad amorphous Ni peak at 45°, which shifted to sharper crystalline peaks after post-heat treatment, indicating the formation of crystalline Ni_3_P and Ni_2_P phases. Similarly, NCF-T samples also showed a transformation from amorphous to crystalline phases, with peak intensities increasing as the temperature was raised to 800 °C, confirming the improvement in crystallinity.

### 2.5. Electrical Conductivity

The electrical resistivities of NGF-T, NGF-T-400, NCF-T, and NCF-T-800 were measured using a Loresta GP resistivity meter (MCP-T610, Mitsubishi Chemical Co., Tokyo, Japan) connected to a four-point probe (MCP-TP03P, Mitsubishi Chemical Co., Japan). To ensure accuracy and consistency, a minimum of 10 measurements were taken for each sample at room temperature (25 °C), and the average resistivity was calculated.

### 2.6. EMI-SE

EMI shielding measurements of the NGF-T/epoxy and NCF-T/epoxy composites were performed in the 500–1600 MHz band at room temperature using a vector network analyzer (VNA, E5062A/EM2107A, Agilent Technologies, Almelo, The Netherlands) with a transmission-reflection mode that complied with ASTM D4935-89 [38]. The EMI-SE was evaluated by measuring the attenuation or reduction of electromagnetic waves (*dB*) using Equation (1).

(1) $$EMI-SE\left(dB\right)=SE_{T}=10\log\frac{P_{1}}{P_{2}}=SE_{R}+SE_{A}+SE_{M}$$

Here, *P*_1_ represents the incident power, *P*_2_ represents the transmitted power, and *SE_R_*, *SE_A_*, and *SE_M_* represent the shielding effects of reflection, absorption, and multiple reflections, respectively. If *SE_A_* > 10 dB [39,40,41], then *SE_M_* can be considered as an absorption loss because most of the re-reflected waves are absorbed by the material by the material. Therefore, Equation (1) can be rewritten as follows:

(2) $$SE_{T}\left(dB\right)\approx SE_{R}+SE_{A}$$

(3) $$SE_{R}\left(dB\right)=10\log\frac{1}{1-R}$$

(4) $$SE_{A}\left(dB\right)=10\log\frac{1-R}{T}$$

*R*, *T*, and *A* are the power coefficients of reflectance, transmittance, and absorbance, respectively, which can be calculated from the four scattering parameters (*S*: *S*_11_, *S*_12_, *S*_21_, *S*_22_) obtained through the VNA measurements.

Here, *R*, *T*, and *A* are the power coefficients of reflectance, transmittance, and absorbance, respectively, which can be calculated from the four scattering parameters (*S*: *S*_11_, *S*_12_, *S*_21_, *S*_22_) obtained through the VNA measurements.

(5) $$R={\left|S_{11}\right|}^{2}={\left|S_{22}\right|}^{2}$$

(6) $$T={\left|S_{12}\right|}^{2}={\left|S_{21}\right|}^{2}$$

(7) A=1−R−T

Here, *S*_11_ and *S*_22_ represent the input and output reflections, respectively, and *S*_12_ and *S*_21_ represent the reverse and forward transmissions, respectively.

### 2.7. Mechanical Test

To evaluate the effects of post-heat treatment on the tensile strength and interfacial shear strength (IFSS) of the Ni-plated fibers (NGF-T and NCF-T), a universal testing machine (LR5K Plus, LLOYD, Bognor Regis, UK) was utilized. The tensile strength was measured using single-fiber tensile testing, following the guidelines of ASTM C 1239-07 [42]. The gauge length of the fiber was set to 25 mm, and the crosshead speed was maintained at 1 mm/min. Each sample was tested at least 20 times, and the average tensile strength was calculated to ensure statistical reliability.

**Figure 2:**
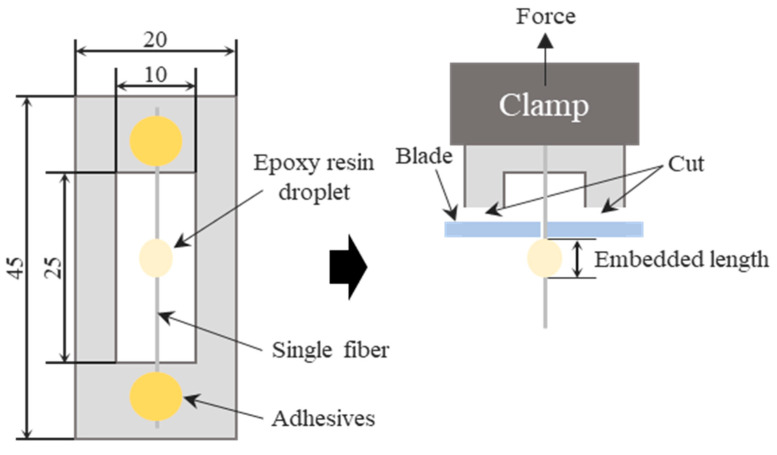
Schematic representation of microdroplet test.

The IFSS of both Ni-coated glass fibers (NGF-T) and Ni-coated carbon fibers (NCF-T) was determined using a microdroplet test, which allows for precise evaluation of the fiber-matrix interfacial bonding strength. The microdroplet specimen preparation method is illustrated in Figure 2. Single NGF-T and NGF-T-400 fibers, as well as NCF-T and NCF-T-800 fibers, were aligned along the centerline of a paper frame, with both ends secured using epoxy resin. An epoxy-resin droplet (80–150 µm in diameter) was deposited at the center of the fiber using a needle, followed by curing at 160 °C for 1 h in an oven to ensure proper adhesion and matrix formation. After curing, the microdroplet specimen was carefully clamped, ensuring that the epoxy-resin droplet was positioned directly under the blade. Both sides of the paper frame were cut to release the fiber, allowing for tensile testing under controlled conditions.

The test speed was set to 0.1 mm/min, and at least 20 measurements per sample were recorded to ensure reproducibility and accuracy. The IFSS was calculated using the following Equation (8):

(8) $$\tau =\frac{F}{\pi DL}$$

Here, *τ* represents the IFSS, *F* represents the peak pull-out force (N), D represents the fiber diameter (µm), and L represents the embedded length of the microdroplet.

## 3. Results and Discussion

### 3.1. Surface Morphology

**Figure 3:**
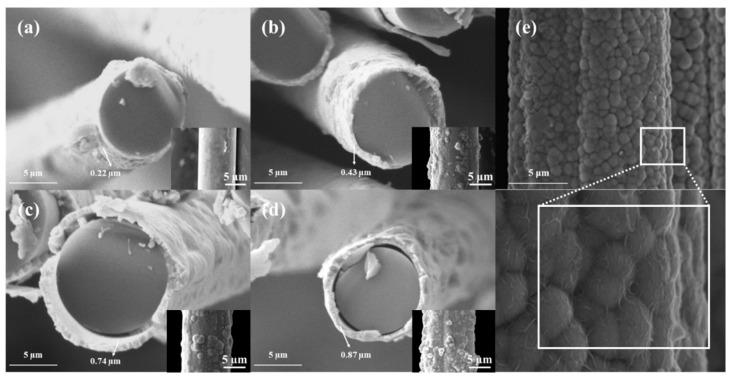
SEM images of Ni-plated fibers at different plating times: (**a**) NGF-1; (**b**) NGF-3; (**c**) NGF-5; (**d**) NGF-10; (**e**) NCF-3, showing progressive increases in Ni layer thickness and surface roughness with plating time, while NCF exhibits more uniform and continuous deposition [35].

**Figure 4:**
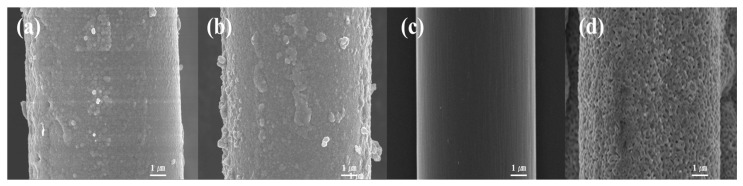
SEM images of NGF before and after post-heat treatment: (**a**) NGF-3; (**b**) NGF-3-400; (**c**) NCF-3; (**d**) NCF-3-800. NGF-3 showed reduced fiber diameter after treatment at 400. NCF-3-800 exhibited clear grain growth and densification of the Ni layer at 800 °C [35].

To evaluate the influence of plating time and post-heat treatment on the surface morphology of Ni-coated glass fibers (NGF-T) and carbon fibers (NCF-T), scanning electron microscopy (SEM) analysis was performed. The corresponding SEM results are presented in Figure 3 and Figure 4. As shown in Figure 3, the NGF-1 sample exhibited poor surface coverage, indicating that a 1-min plating duration was insufficient to achieve a uniform nickel layer. With increasing plating time from 3 to 10 min, the Ni coating thickness progressively increased from 0.22 to 0.87 µm. This was accompanied by greater surface roughness, attributed to the agglomeration and growth of nickel particles. Such morphological evolution contributes to the formation of more continuous and conductive networks, thereby improving the electrical conductivity and EMI shielding effectiveness (EMI-SE) of the composite.

In contrast, the NCF-T samples exhibited a relatively smooth and continuous Ni layer even at shorter plating durations. This is likely due to the higher surface energy and better wettability of carbon fibers compared to glass fibers, which facilitates more uniform electroless deposition. Following post-heat treatment at 800 °C, the NCF-T-800 samples showed grain coarsening and densification, indicative of a transformation from amorphous to crystalline phases, as further confirmed by XRD analysis [35].

For NGF-T samples, SEM images (Figure 4) revealed that post-heat treatment at 400 °C did not significantly alter surface morphology. However, a reduction in average fiber diameter from 9.60 to 9.29 µm was observed. This dimensional change is attributed to volumetric shrinkage during the transformation of amorphous Ni to crystalline Ni_3_P, which typically occurs around 350 °C [43]. Crystalline phases are known to exhibit higher density than their amorphous counterparts [44], and such phase transitions are frequently accompanied by structural densification and shrinkage. These findings suggest that heat treatment effectively induced crystallization of the nickel layer, promoting densification and compaction of the coating structure.

### 3.2. Structural Properties

**Figure 5:**
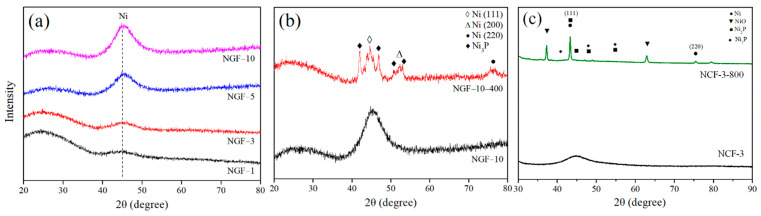
XRD patterns of Ni-plated fiber composites: (**a**) NGF samples with varying plating times, showing increased amorphous Ni peak intensity near 45°; (**b**) NGF-10 before and after heat treatment at 400 °C, with crystalline Ni and Ni_3_P peaks emerging post-treatment; (**c**) NCF-3 before and after heat treatment at 800 °C, showing phase transformation to Ni, NiO, Ni_2_P, and Ni_3_P [35].

X-ray diffraction (XRD) analysis was performed to examine the crystallographic evolution of NGF-T and NCF-T before and after post-heat treatment. The corresponding XRD patterns are shown in Figure 5. Figure 5a displays the XRD patterns of NGF samples as a function of plating duration. All samples exhibited a broad diffraction peak near 2θ ≈ 45°, characteristic of amorphous Ni. As plating time increased, the intensity of this peak also increased, reflecting a progressive accumulation of Ni on the fiber surface. This trend is consistent with the plating thickness data obtained vis SEM (Figure 1), suggesting that the observed intensity enhancement directly correlates with increased Ni layer thickness. Figure 5b presents the XRD results for NGF-10 before and after post-heat treatment. Thermal processing transformed the initially broad amorphous Ni(111) peak into a sharp crystalline reflection. Additional diffraction peaks corresponding to Ni(200), Ni(220), and Ni_3_P were also observed, indicating the formation of a more ordered Ni structure and associated intermetallic phases. Figure 5c similar XRD transformations for NCF-3 upon heat treatment. The broad Ni(111) signal sharpened into a distinct crystalline peak, accompanied by new reflections attributed to NiO, Ni_2_P, and Ni_3_P. These phase evolutions imply both crystallization and chemical transformation of the Ni layer during thermal exposure.

As previously reported, amorphous Ni begins to crystalize into Ni_3_P at annealing temperatures around 350 °C. The emergence of crystalline Ni_3_P in NGF samples treated at 400 °C confirms this transition pathway. In the case of NCFs, which underwent heat treatment at higher temperatures (800 °C), the formation of additional phases such as Ni_2_P and NiO suggests more extensive thermal activation, likely due to the superior thermal stability of carbon fibers compared to glass fibers. The enhanced sharpness and definition of the XRD peaks following heat treatment reflect increased crystallinity in the plated layers. This structural reorganization is closely associated with improved electrical conductivity EMI-SE. These findings are in agreement with the grain coarsening and densification trends observed in SEM images, further validating the impact of post-heat treatment on microstructure and performance.

### 3.3. Electrical Properties

**Figure 6:**
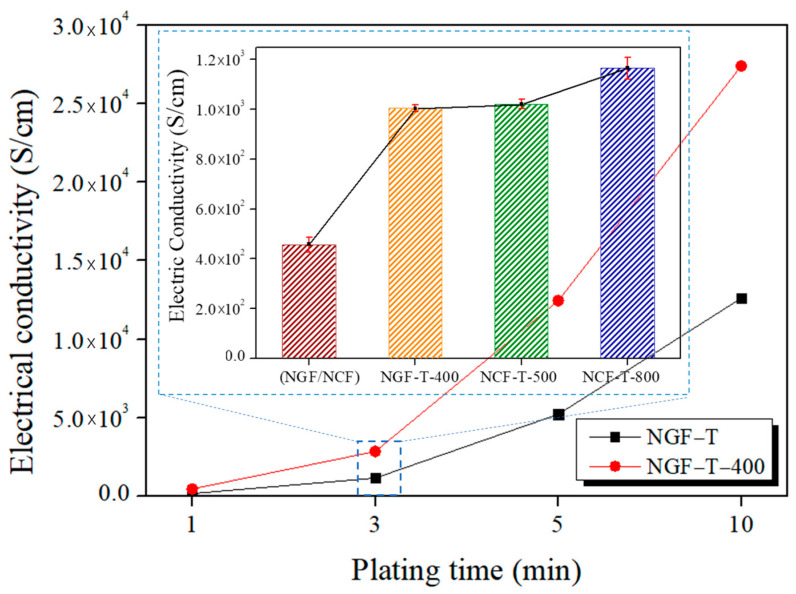
Effects of plating time and post-heat treatment on the electrical conductivity of Ni-plated fibers. Conductivity increases with plating time, and further enhancement is observed after heat treatment due to Ni crystallization. NGF-T-400 shows a 169% improvement, while NCF-T-800 exhibits the highest conductivity [35].

Electrical conductivity is a key parameter influencing the electromagnetic interference shielding effectiveness (EMI-SE) of conductive composites. The electrical conductivities of NGF-T, NGF-T-400, NCF-T-500, and NCF-T-800 were measured using a four-point probe method, and the results are presented in Figure 6. For NGF-T, conductivity increased significantly with plating time, ranging from 1.72 × 10^2^ to 1.26 × 10^4^ S/m. After post-heat treatment at 400 °C, the NGF-T-400 sample exhibited a further enhancement in conductivity to 2.74 × 10^4^ S/cm, representing a 169% improvement over the untreated condition. This enhancement is attributed to the crystallization of the Ni layer, particularly the formation of Ni_3_P from amorphous Ni, which lowers contact resistance and improves electron transport pathways. The XRD patterns shown in Figure 5b support this conclusion, confirming the phase transformation from amorphous Ni to crystalline Ni_3_P upon heat treatment. Since crystalline structures typically exhibit higher electrical conductivities than amorphous counterparts [45], the observed increase in NGF-T-400 is consistent with the structural evolution. In the case of NCF-T, post-heat treatment at 800 °C also led to a conductivity increase from 4.0 × 10^2^ to 1.2 × 10^3^ S/cm. Despite the lower absolute values compared to NGF-T-400, the superior electrical performance of NCF-T-800 is attributed to the intrinsic advantages of carbon fibers, such as enhanced crystallinity, structural integrity at high temperatures, and improved electron mobility [35].

### 3.4. EMI Shielding Properties

**Figure 7:**
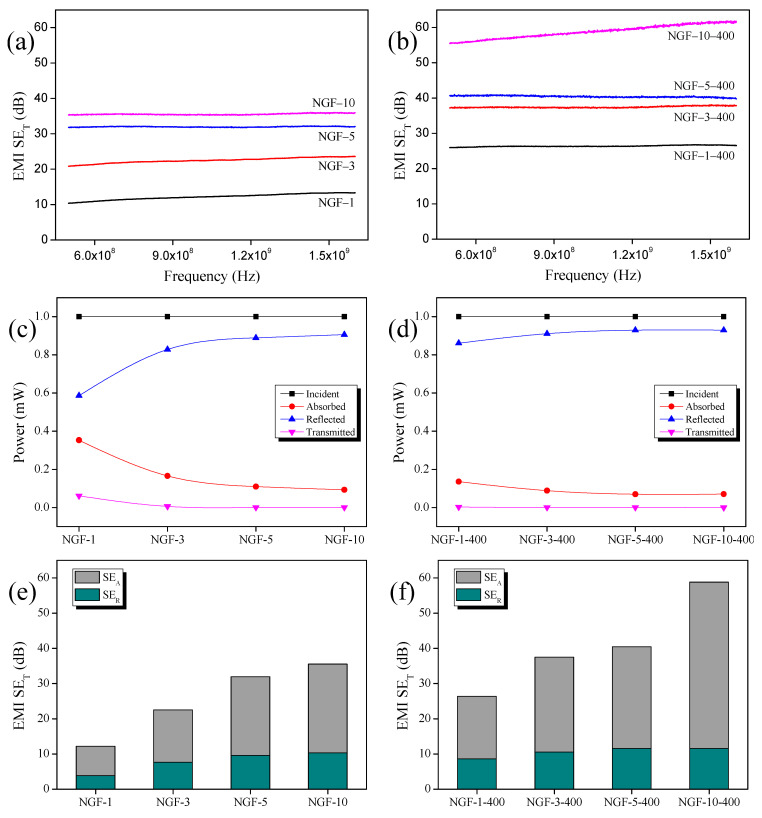
(**a**,**b**) EMI-SE of NGF-T/epoxy and NGF-T-400/epoxy composites with different Ni-plating times; (**c**,**d**) power coefficients A, R, and T of NGF-T/epoxy and NGF-T-400/epoxy composites fabricated with different Ni-plating times; (**e**,**f**) SE_T_ expressed as the sum of the average values of SE_R_ and SE_A_ for NCF-T/epoxy and NCF-T-400/epoxy composites prepared with different Ni-plating times.

The EMI-SE of the NGF-T/epoxy composites was evaluated over the frequency range of 500–1600 MHz, and the results are shown in Figure 7a,b. As plating time increased, the total shielding effectiveness (SE_T_) values of NGF-T composites also improved. The NGF-T-400/epoxy samples exhibited higher SET values than NGF-T counterparts plated for the same duration, showing enhancements of 26% to 116%, depending on the plating time. Notably, the NGF-3-400/epoxy composite outperformed the NGF-10/epoxy composite despite having undergone a shorter plating time. This result suggests that post-heat treatment plays a dominant role in improving EMI-SE by promoting structural densification and enhancing electrical conductivity, as previously observed in microstructural and electrical analyses.

To further analyze the shielding behavior, the average values of absorption (A), reflection (R), and transmission (T) coefficients were calculated from the S-parameters, as shown in Figure 7c,d. In both NGF-T and NGF-T-400 composites, R increased while A decreased with increasing plating time. This trend reflects a growing impedance mismatch between the material surface and free space, which results in greater initial reflection of incident electromagnetic waves. Absorption occurs subsequently within the material due to conduction, dielectric, and magnetic losses [46]. The increased reflection (R) observed in NGF-T-400 is consistent with its higher electrical conductivity (Figure 7), which further amplifies impedance mismatch and reduces the number of waves penetrating the material [47]. Consequently, the NGF-T-400 composite exhibited higher reflection (R) and lower absorption (A) than the corresponding NGF-T samples.

Figure 7e,f show the SE_T_ values as the sum of shielding by absorption (SE_A_) and shielding by reflection (SE_R_). Unlike the power coefficients A and R, SE_A_ and SE_R_ quantify the energy-based shielding performance, representing the intrinsic potential of the material to attenuate electromagnetic energy. In both NGF-T and NGF-T-400 composites, SE_A_ and SE_R_ increased with plating time, with SE_A_ contributing more strongly to overall SE_T_.

However, SE_A_ and SE_R_ trends differ from those of the power coefficients. Although A was consistently lower than R, SE_A_ exceeded SE_R_, which can be attributed to the different physical bases of the respective coefficients. A, R, and T are calculated from the power of the incident waves, while SE_A_, SE_R_, and SE_T_ are based on the energy absorbed and reflected by the material [14]. This distinction often leads to misinterpretation of dominant shielding mechanisms. Therefore, as noted by recent studies [48,49], a comprehensive analysis of the shielding mechanism requires first evaluating macroscopic behavior using A and R, followed by a more detailed interpretation using SE_A_ and SE_R_. Comparing SE_A_ and SE_R_ alone does not fully capture the wave–material interaction dynamics.

The NGF-T-400 composite achieved a maximum EMI-SE of 90 dB, representing up to a 116% increase over the untreated NGF-T. In comparison, the NCF-T-800 composite exhibited an even higher EMI-SE of 110 dB, attributed to its superior intrinsic conductivity and structural stability following high-temperature treatment. These results confirm that both post-heat treatment and fiber type play critical roles in optimizing EMI shielding performance.

### 3.5. Mechanical Properties

**Figure 8:**
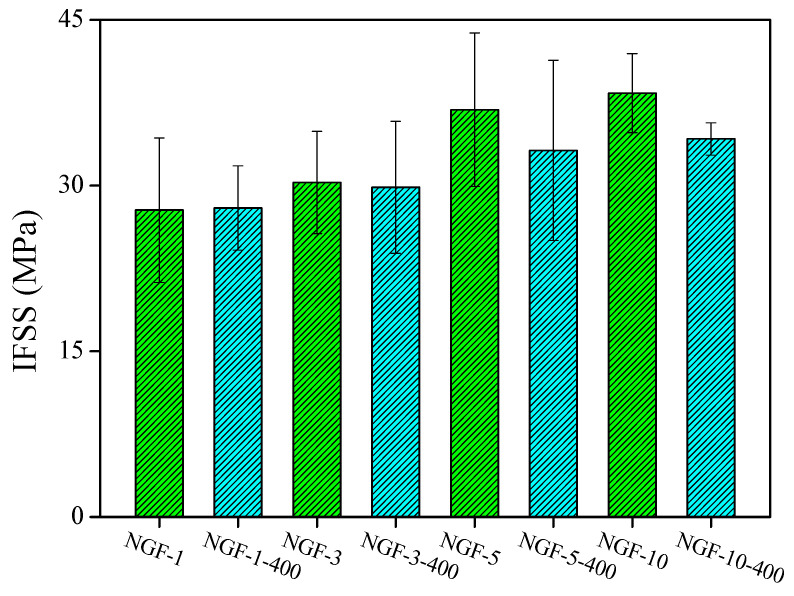
Interfacial shear strength (IFSS) of Ni-plated glass fibers as a function of plating time and post-heat treatment. IFSS increases with plating time due to the thickened and roughened Ni layer, which enhances mechanical interlocking, while post-heat treatment leads to a reduction in IFSS, likely due to volumetric shrinkage and brittle Ni_3_P formation.

**Figure 9:**
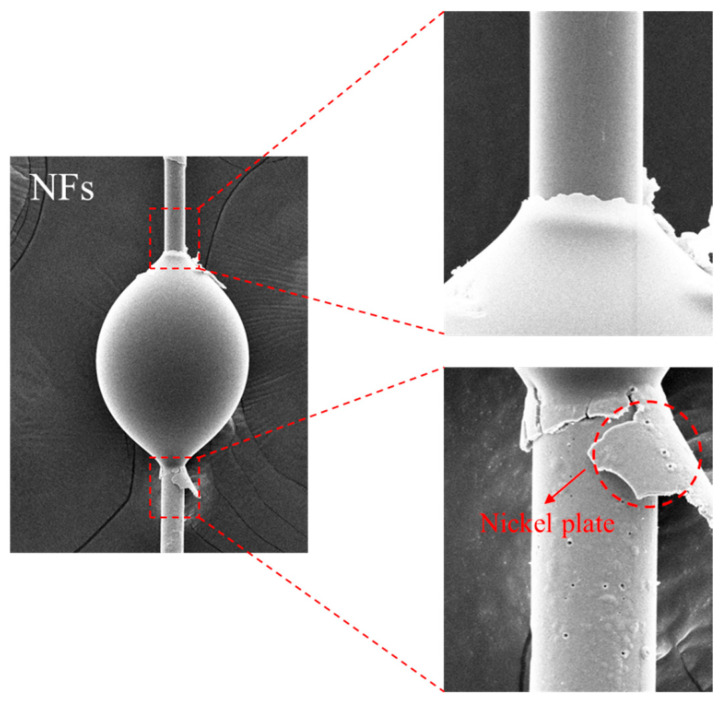
Fracture morphology of Ni-plated fibers after the IFSS test. Debonding and Ni layer fracture are observed, indicating that plating thickness and adhesion affect interfacial failure behavior.

The interfacial shear strength (IFSS) was measured before and after post-heat treatment to evaluate the mechanical integrity of the fiber–matrix interface. The results are shown in Figure 8 and Figure 9. As plating time increased, the IFSS values of both NGF-T and NCF-T samples improved.

This enhancement is attributed to the increased thickness and surface roughness of the Ni coating, which promotes stronger mechanical interlocking at the interface. The increase in surface roughness effectively enlarges the specific surface area, thereby improving stress transfer across the fiber–matrix boundary. Additionally, as shown in Figure 9, the thickened Ni layer requires greater force to fracture during interfacial failure, further contributing to the increase in IFSS.

However, after post-heat treatment, NGF-T-400 samples exhibited a reduction in IFSS compared to untreated samples with equivalent plating times. This decrease is attributed to volumetric shrinkage arising from the mismatch in thermal expansion coefficients between GF/Ni and Ni/epoxy interfaces, particularly at elevated temperatures (e.g., 400 °C for NGF-T) [50,51]. Furthermore, the formation of brittle intermetallic phases such as Ni_3_P during heat treatment is believed to reduce interfacial strength [52].

In the case of NCF-T-800, the IFSS reduction was more pronounced, which may be attributed to the higher heat-treatment temperature (800 °C), promoting the formation of Ni_2_P, a phase known for its mechanical brittleness and limited bonding capability [35]. These results indicate that while Ni plating enhances interfacial bonding, post-heat treatment must be carefully controlled to avoid adverse effects from brittle phase formation and thermal mismatch.

## 4. Conclusions

The increasing integration of electronic systems and wireless communication technologies into modern infrastructure has intensified concerns regarding EMI, emphasizing the need for advanced shielding materials with high electrical conductivity and structural reliability. To address this challenge, the present study investigated the use of electroless Ni plating on fiber reinforcements as a strategy to enhance both electrical and mechanical performance in composite systems. The effects of post-heat treatment on the EMI-SE and IFSS of Ni-plated fiber/epoxy composites were systematically evaluated.

Electroless Ni plating significantly improved the surface conductivity and roughness of GFs, leading to enhanced EMI shielding and interfacial adhesion. Post-heat treatment at 400 °C induced a transformation of the amorphous Ni layer into crystalline Ni_3_P, resulting in a 169% increase in electrical conductivity. However, this crystallization also led to microstructural densification and the formation of brittle intermetallic phases, which contributed to a reduction in IFSS. Despite this trade-off, NGF-T-400 composites demonstrated up to a 116% increase in EMI-SE compared to non-treated counterparts.

To provide comparative insights, Ni-plated carbon fibers NCF-T were also examined. Heat treatment at 800 °C promoted the formation of Ni_2_P and Ni_3_P phases in NCF-T, leading to significant improvements in electrical conductivity (1.0 × 10^3^ S/cm) and the EMI-SE (up to 110 dB) while maintaining excellent thermal and structural stability. These results underscore the superior shielding performance of NCF-T, particularly in high-frequency environments, whereas NGF-T offers a cost-effective alternative for cementitious applications, such as smart concrete and EMI-shielding construction materials.

Overall, the findings of this study demonstrate that both Ni-plated fibers NGFs and NCFs represent a viable and scalable solution for developing EMI shielding composites that also retain mechanical integrity. Their integration into smart infrastructure components, including high-frequency communication hubs, data centers, and EMI-sensitive civil structures, holds substantial promise for multifunctional performance.

Despite the favorable results, further research is warranted to optimize interfacial bonding and mitigate the mechanical degradation associated with post-heat treatment. Additionally, the exploration of alternative metallization strategies and advanced surface modification techniques could further tailor the electromagnetic and mechanical performance of these systems.

In conclusion, the post-heat treatment of Ni-plated fiber composites offers a practical route to achieving high EMI shielding effectiveness with preserved mechanical properties, contributing to the development of next-generation intelligent infrastructure, smart concrete, and lightweight EMI-shielding enclosures suited for modern construction and urban environments.

## Figures and Tables

**Table 1:** Composition and condition of Ni-loaded baths.

Category	Electroless Plating	Value
Composition	NiSO_4_·6H_2_O (g/L)	280
	NiCl_2_·6H_2_O (g/L)	40
	Na_3_C_6_H_5_O_7_·1.5H_2_O (g/L)	15
	NaH_2_PO_2_·2H_2_O (g/L)	100
	NH_4_Cl (g/L)	100
	PbNO_3_ (g/L)	30
Condition	pH	8.25
	Temperature (°C)	85 ± 2
	Time (min)	1, 3, 5, and 10

## Data Availability

The original contributions presented in this study are included in the article. Further inquiries can be directed to the corresponding author(s).
